# Preliminary reliability of South African adaptation and Northern Sotho translation of the Modified Checklist for Autism in Toddlers, Revised with Follow-Up

**DOI:** 10.4102/sajcd.v68i1.831

**Published:** 2021-07-22

**Authors:** Carlien Vorster, Alta Kritzinger, Lovina E. Coetser, Jeannie van der Linde

**Affiliations:** 1 Department of Speech-Language Pathology and Audiology, Faculty of Humanities, University of Pretoria, South Africa; 2 Department of Statistics, Faculty of economics and Business Management, University of Pretoria, South Africa

**Keywords:** autism screening, M-CHAT-R/F-Northern Sotho translation, preliminary reliability, low and middle-income country, South African adapted English M-CHAT-R/F

## Abstract

**Background:**

There is a shortage of validated autism screening tests in the 11 official languages of South Africa. The Modified Checklist for Autism in Toddlers, Revised with Follow-Up (M-CHAT-R/F^TM^), a validated and well-known screening test, had already been adapted (in English) and translated into Northern Sotho for use in South Africa.

**Objectives:**

The aim was to collect pilot data to determine the preliminary reliability and feasibility of the two tests to confirm the equivalence of the adaptation and translation.

**Method:**

The study was conducted in a peri-urban community in South Africa. Twenty-one first-language Northern Sotho caregivers of children aged between 18 and 48 months were recruited by employing snowball sampling. The participants were asked to complete the Northern Sotho and the culturally adapted English M-CHAT-R/F, which were presented in random order.

**Results:**

The preliminary content validity and equivalence were evident, with no difference at the 5% interval of the Wilcoxon signed rank test. All 21 toddlers screened presented with a low risk for autism following the recommended execution of the Follow-Up section for the toddlers in the medium risk category. All participants completed the two screening tests, with none indicating unfamiliar words or constructs. A higher preference for the English adapted version was found but a need for the Northern Sotho screening test was also evident

**Conclusion:**

The Northern Sotho translation of the M-CHAT-R/F, as well as the adapted English version, appears feasible and is ready for comprehensive validation.

## Introduction

The lack of culturally appropriate screening instruments for autism has become a universal concern (Hyman, Levy, Myers, & AAP Council on Children with Disability, 2020; Malcolm-Smith et al., 2013). Most autism screening tools are available in English only, as they derive from English-speaking countries (Soto et al., 2015). Cultural and linguistic differences in the understanding of test items and concepts are some of the factors that may lead to disparities in screening outcome (Barton, Dumont-Mathieu, & Fein, 2012; Soto et al., 2015). In an attempt to address the shortage of validated, cultural and linguistic appropriate screening tools, and amidst a worldwide steady increase in the prevalence of autism (Maenner et al., 2020), the authors had previously adapted and translated one of the most commonly used autism screening tests for use in South Africa (Vorster et al., 2021).

Limited research has been performed to develop and validate screening instruments on the African continent (De Vries, 2016; Franz, Chambers, von Isenburg, & de Vries 2017). In a multicultural and multilingual country such as South Africa, local translation and validation of autism screening tools are important (Franz et al., 2018). Early detection of developmental conditions is a high priority and advocated by the World Health Organisation (WHO), because identification at a young age may decrease the impact of impairments as it promotes early management (WHO, 2013a).

The original English Modified Checklist for Autism in Toddlers, Revised with Follow-Up (M-CHAT-R/F^TM^) (Robins et al., 2014) was adapted and translated into Northern Sotho. The International Test Commission (ITC, 2017) and WHO (2013b) guidelines were used. A rigorous translation and adaptation methodology, which involves cultural adaptation, forward and back translation, has become well established in recent years (ITC, 2017). A multidisciplinary specialist panel reviewed the test after a double translation procedure. The comprehensive process resulted in two versions of the original test, a South African culturally adapted English version as well as a culturally appropriate Northern Sotho translation of the M-CHAT-R/F (Vorster et al., 2021). Test translation without cultural adaptation may ignore item bias and may therefore contribute to invalid screening outcomes (ITC, 2017).

The value of a screening test in an indigenous African language and an adapted English version was shown by Van der Merwe et al. (2017). The study investigated the language preference of isiZulu-speaking parents of two versions of a developmental screening tool, the Parents’ Evaluation of Developmental Status [PEDS] (Glascoe, 2013), in a peri-urban community. The results showed that 54% of the isiZulu-speaking participants preferred the English version of the PEDS, whereas 46% preferred the isiZulu translation. This finding demonstrates that both the English as well as the indigenous language versions are accepted and desired in South Africa, as English is considered an urban language (Posel & Zeller, 2016).

Apart from variation in the language preference of caregivers who complete a screen, it is also important to consider cultural variability in the perception of child behaviour. Differences in the perception of behaviour may influence screening outcomes (Barton et al., 2012; Soto et al., 2015). As most parent-completed questionnaires are based on observed child behaviour, a clear rationale is evident for the cultural adaptation of instruments. To promote fairness in testing, screening tools need to be developed for populations that are not first-language English speakers (Hyman et al., 2020). The M-CHAT™ has already gone through a rigorous revision process to simplify the language for greater comprehensibility, making it an ideal screening test to translate (Robins et al., 2014).

Numerous translations and/or adaptations of the M-CHAT™ and M-CHAT-R/F™ resulted in 67 different versions of the instrument (Robins, Fein, & Barton, 2018). An example of such an adaptation and translation was carried out by Brennan, Fein, Como, Rathwell and Chen (2016). The authors developed an Albanian version of the M-CHAT-R/F (M-CHAT-R-A) by translating the instrument and removing three test items. The omission of items improved the positive predictive value, supporting the need for test adaptation for a specific setting. A systematic review of cultural adaptation and translation of autism-specific screening instruments found that rigorous adaptation and translation often result in more modifications such as adding cultural appropriate information and/or behavioural examples, employing alternative words and constructs (Soto et al., 2015).

With the current adaptation of the screen for South African users, unfamiliar cultural constructs were identified in word use, interpretation and descriptions of child behaviour. Four changes were made to the M-CHAT-R/F™. The first involved a child’s eye contact when communicating with a caregiver. Making direct eye contact with superiors is inappropriate in various Southern African cultures (Mncwango, 2009). The item was thus adapted to ‘Does your child look in *your direction* or in the eye when you are talking to them?’. ‘Make-believe’, ‘soft toys’ and ‘playground equipment’ were also identified as unfamiliar constructs in Northern Sotho culture and were adapted to ‘acting’, ‘toys’ and ‘trees’, respectively. These items read: ‘If you point at something across the room, does your child look at it? (For example, if you point at a *toy* or an animal, does your child look at *the toy* or animal?)’; ‘Does your child *act*?’ and ‘Does your child like climbing on things? (For example, furniture, *trees*, or stairs)’, respectively. The greatest challenge with the translation of the M-CHAT-R/F™ was ensuring accurate and equivalent translation of the technical content of the test administration instructions.

The two versions of the M-CHAT-R/F were available to be tested by the intended users, that is, Northern Sotho-speaking caregivers in South Africa. The aim of the study was to collect pilot data that allowed item analysis, assessment of the preliminary reliability, and degree of agreement between the two test versions. A second aim was to describe the referral rate of the adapted and translated versions. Lastly, caregivers’ preference of the two versions of the test was investigated. If any discrepancies between the tests or difficulties were shown by the results, adjustments could have been made before further validation with a large sample. A descriptive comparative design was employed to achieve the study aims.

## Methods

### Participants

A total of 21 participants, living in a peri-urban community in Gauteng, South Africa, were selected with snowball recruiting. The first point of contact was two active community residents known to the researcher. These residents identified families with toddlers aged between 18 and 48 months, with no diagnosed conditions, at a community church and a day care centre. Participants were first-language Northern Sotho-speaking mothers and grandmothers of 18- to 48-month old toddlers. The M-CHAT-R/F™ was initially developed for toddlers between the ages of 18 and 30 months. Yama et al. (2012) however, found that the M-CHAT-R/F™ is relevant for children up until 48 months of age. Similar to the requirements to complete the original M-CHAT-R/F™, participants had to have passed Grade 4 and be able to read Northern Sotho or Sepedi^1^ and English. Participants were excluded from the study if their toddler had been diagnosed with conditions such as a sensory deficit (e.g. hearing loss), a genetic syndrome or cerebral palsy. Using the Road to Health Booklet developmental screen and parental report, the aim was to exclude toddlers with developmental conditions whilst including typically developing children in the reference population.

The culturally adapted English M-CHAT-R/F, as well as the Northern Sotho translated M-CHAT-R/F were used as screening instruments. A socio-demographic questionnaire was included to allow for comprehensive sample description (Table 1). Following the completion of the 20 questions of each version of the M-CHAT-R/F, participants were requested to complete the caregiver feedback form which included three questions about the test: (1) ‘Do you prefer to answer the test in English or Northern Sotho/Sepedi?’; (2) ‘Were there any words that you do not know? If yes, please list the words’; (3) ‘Were there any items in the M-CHAT-R/F that you did not really understand? If yes, please mark the items’.

**TABLE 1:** Participant characteristics (*n* = 21).

Participant characteristics	Variable	*n*	%	Mean (SD)	Mode
Additional language	English	21	100.00	-	English (*n* = 21); 100%
	Xitsonga	4	4.76	-	-
	Setswana	2	9.52	-	-
	isiZulu	1	19.05	-	-
Gender of child	Female	18	85.7	-	Female (*n* = 18); 58.7%
	Male	3	14.29	-	-
Age of child	18–23 months	5	23.80	29 months (9 months)	18 months (*n* = 4); 19.05%
	24–35 months	8	28.10	-	24 months (*n* = 4); 19.05%
	36–48 months	8	38.10	-	36 months (*n* = 4); 19.05%
Age of participants	18 years	2	9.52	30 years and 8 months	Age category 31 ≤ 34 (*n* = 7); 33.33%
	19–22 years	0	0.00	-	-
	23–26 years	3	14.29	-	-
	27–30 years	4	19.05	-	-
	31–34 years	7	33.33	-	-
	35–40 years	4	19.05	-	-
	46–50 years	1	4.76	-	-
Participant education	Grade 9	4	19.05	Grade 12 (National Senior Certificate)	Grade 12 (National Senior Certificate) (*n* = 14); 66.67%
	Grade 12	14	66.67	-	-
	Degree	2	9.52	-	-
	Not specified	1	4.76	-	-
Social support grant for child	Yes	16	76.19	-	Yes, receives a grant (*n* = 16); 76.19%
	No	5	23.81	-	-

SD, standard deviation.

Following institutional ethical clearance, participants were required to provide written informed consent. The two versions of the M-CHAT-R/F were presented in a random order to participants. Eleven participants completed the Northern Sotho translation first and the remaining 10 completed the English adaptation first. The random presentation controlled for a learning effect to ensure reliability of data. When a toddler was identified as being at medium risk for autism, the Follow-Up section of the instrument was conducted telephonically afterwards as per M-CHAT-R/F™ instructions. No high-risk cases were identified.

### Data analysis

Both screening instruments were scored according to the existing test instructions, to determine the child’s risk for autism. Questions 2, 5 and 12 require ‘No’ or ‘Aowa’ as the negative screen. For the remaining items ‘Yes’ or ‘Ee’ was deemed an accurate answer for a negative screen. For each answer deviating from the prescribed norm, a score count of 1 was allocated. Following the allocation of 0 or 1, the sum of the score was determined, whereafter the risk category was identified. Three risk categories for autism are indicated in the test. Low-risk occurs when a score between 0 and 2 is obtained, medium risk is a score between 3 and 7 and a high-risk score is more than 8. If a toddler obtains a medium-risk score, the Follow-Up section of the M-CHAT-R/F should be completed following the initial completion of the screen. If a high-risk score is identified, a child should be referred to a medical professional immediately.

The two sets of completed test items were compared to determine inconsistencies in the participants’ answers. Descriptive statistics were used to describe the population and the percentage agreement between the two versions. The non-parametric test was employed to determine if there were any significant differences between the two caregiver-completed test versions. Non-parametric statistical analysis was used because of the small sample size employed in the pilot study. Wilcoxon signed rank test was used to determine agreement between test items of the two versions, supporting the preliminary reliability. The risk profiles were analysed to describe the referral rate of the two versions. Data were further interpreted to determine which items were not completed, not understood or required clarification.

### Ethical considerations

The study was approved by the University of Pretoria, Faculty of Humanities Research Ethics Committee on 11 November 2019 (reference number: 29026319 [HUM041/0919]). The authors have permission from Diana Robins to adapt, translate and validate the M-CHAT-R/F™. All the participants signed an informed consent letter that has been approved by the Institutional Research Board. As part of the informed consent, the participants provide consent for the anonymous use of the data collected in scientific papers.

## Results

**FIGURE 1:**
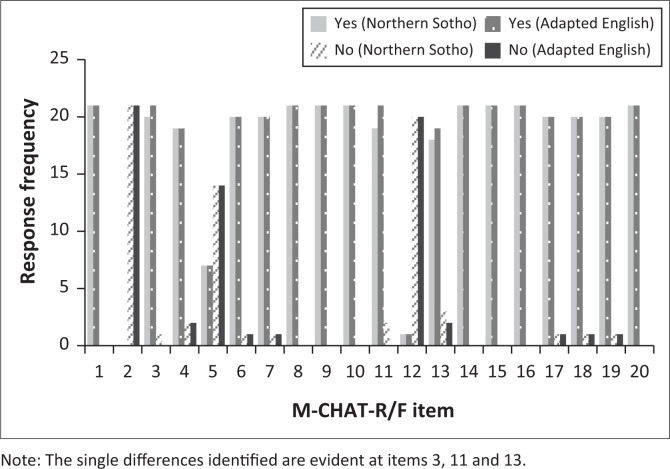
Comparison between Northern Sotho versus adapted English Modified Checklist for Autism in Toddlers, Revised with Follow-Up (M-CHAT-R/F).

**FIGURE 2:**
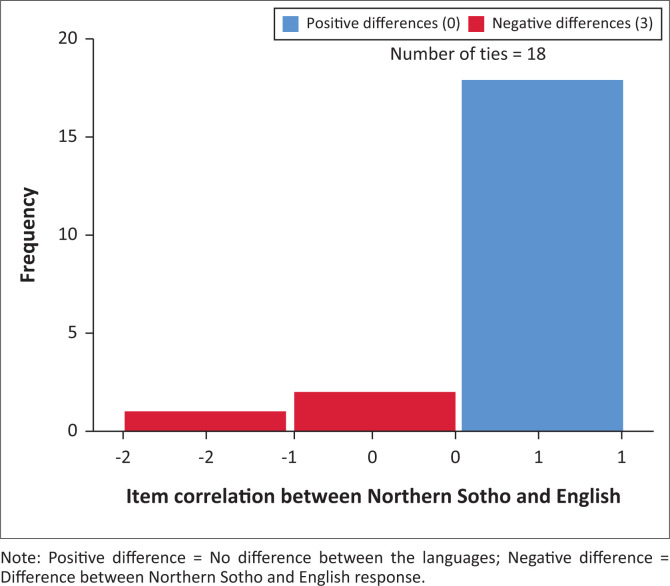
Wilcoxon signed rank test results with item correlation between English adapted version and Northern Sotho version of the Modified Checklist for Autism in Toddlers, Revised with Follow-Up.

The 21 data sets represented 420 pairs of completed test items. Two participants showed a single response difference, answering ‘Yes’ to a specific question in the one screening test and ‘No’ in the other. A third participant had two items with a difference in answers. This difference resulted in 416 pairs (99%) yielding an equivalent answer and four pairs (1%) presenting differing answers. The differences are evident in Table 2 and Figure 1. In Figure 2, this difference is evident with 18 data sets having no difference and three data sets presenting with ‘a negative difference’.

**TABLE 2:** Response frequency for Northern Sotho and adapted English Modified Checklist for Autism in Toddlers, Revised with Follow-Up.

Question	Yes (Northern Sotho)	Yes (Adapted English)	No (Northern Sotho)	No (Adapted English)	% agreement
1	21	21	0	0	100
2	0	0	21	21	100
3	**20**	**21**	**1**	**0**	**95**
4	19	19	2	2	100
5	7	7	14	14	100
6	20	20	1	1	100
7	20	20	1	1	100
8	21	21	0	0	100
9	21	21	0	0	100
10	21	21	0	0	100
11	**19**	**21**	**2**	**0**	**90**
12	1	1	20	20	100
13	**18**	**19**	**3**	**2**	**95**
14	21	21	0	0	100
15	21	21	0	0	100
16	21	21	0	0	100
17	20	20	1	1	100
18	20	20	1	1	100
19	20	20	1	1	100
20	21	21	0	0	100

Note: The items in bold indicate the three items with a difference in response between the two versions.

The Wilcoxon signed rank test was used to determine item correlation between the two versions. Despite only three item differences in participant answers between the English and Northern Sotho versions, the Wilcoxon signed rank test identified no difference between the two versions of the M-CHAT-R/F scores at a 5% level, with a score of 0.102. This provides preliminary evidence of near-perfect agreement and reliability of the two versions. Figure 2 indicates the agreement between the two versions and depicts three differences between the English and Northern Sotho versions.

The two items showing a once-off difference in two different data sets were items 3 and 13. Two of the three identified participants additionally presented with a difference when answering item 11. In all instances, participants gave a ‘Yes’ answer in English and a ‘No’ answer in Northern Sotho. In two of the three cases, the English version was completed first. For instance, a participant indicated ‘Yes’ that the toddler smiles back when the caregiver smiles at her, but in the Northern Sotho version she stated ‘No’ it does not happen. Another example shows ‘Yes’, the child can walk and in the Northern Sotho version ‘No’ the child does not walk. In the case history completed by the participants, no delayed milestones were identified. None of the participants indicated that they had any difficulty understanding words or concepts with no underlying pattern in the errors evident.

**FIGURE 3:**
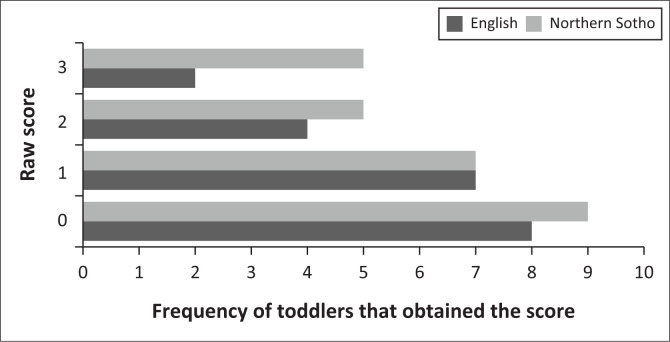
Risk profile according to the raw score for each version of the Modified Checklist for Autism in Toddlers, Revised with Follow-Up.

Similar risk profiles of the toddlers were found in the two versions of the test as evident in Figure 3. The mean risk-score of the adapted version was 0.810, (σ = 0.814) (total raw score of 0–2 indicates low-risk for autism). No Follow-Up questions were therefore necessary. The mean risk-score for the Northern Sotho version, before completing the Follow-Up questions, was 1.0 (σ = 1), also low-risk. As evident in Figure 2, the Northern Sotho Follow-Up questions were required for two participants whose children scored in the medium-risk category (total raw score between 3 and 7), whilst no participants required follow-up from the adapted version. The Follow-Up questions were items 5, 11 and 13 in both sets, respectively. After completion of the Follow-Up questions, the two toddlers showed low-risk profiles.

Although no risk for autism, the children showed some developmental risks such as pre-term birth with a gestational age lower than 36 weeks, low birth weight and APGAR scores below 5. The APGAR score is a universally used new-born health assessment considering the infant’s muscle tone, heart rate, reflex, respiratory effort, irritability and colour (Cnattingius et al., 2017).

Of the 21 participants, only 8 (38%) indicated that they prefer the Northern Sotho version and 13 (62%) stated that they would rather complete the English adapted version of the screening test. The small sample size did not allow for statistical analysis to determine if a correlation exists between the participants’ language preference, age and level of education.

## Discussion

### Key findings

The two South African versions of the M-CHAT-R/F were previously developed by our team (Vorster et al., 2021) and were now piloted with a small sample of Northern Sotho-speaking caregivers. The study aimed to determine the agreement, equivalence and preliminary reliability of the two South African versions of the screening test. Additionally, participants’ understanding of the language and constructs used in the tests, and their test version preference were investigated.

Equivalence between the two test versions, with no difference at the 5% level regarding item correlation, is evident. Linguistic, construct and technical equivalence were shown by comparing the answers to both versions as recommended by DuBay et al. (2021). The absence of variation between the responses to the two versions is an indication that the versions yielded the same answers, confirming the preliminary test-retest reliability of the tests. Both the initial 20 questions as well as specific Follow-Up questions were used in this study. This resulted in comprehensive use of the two test versions. No additional changes are necessary before a large-scale validation study can commence.

The referral rate of the Northern Sotho version, with two children initially showing a medium risk for autism, but low-risk after the Follow-Up questions, was similar to that of the initial validation study of the M-CHAT-R/F™ conducted by Robins et al. (2014). Despite a smaller sample size (*n* = 21), the construct validity of the translated Northern Sotho version appears to be similar to the M-CHAT-R/F™ when comparing the Follow-Up rate. In the current study, a total of 90.4% of the screenings indicated that the toddlers were low risk for autism (screening negative) and (*n* = 2) 9.6% of the toddlers identified required the Follow-Up questions. The two toddlers were 25 and 36 months of age, respectively. The large validation study (*n* = 16 071) identified 92.5% toddlers as screening negative (low-risk) for autism and 7.5% screening positive for a Follow-Up session (Robins et al., 2014). The function of the Follow-Up questions is to provide caregivers with an opportunity for clarification as different examples of behaviour are included to prevent false-positive results. With the completion of the Follow-Up questions, none of the pilot study participants’ toddlers was found to be at risk for autism showing that an additional opportunity to clarify their answers was necessary.

The high number of low-risk cases found in the pilot study is to be expected with a sample size of only 21 despite the global increase in the prevalence of autism. Consistent with the use of snowball sampling, there may have been selection bias, thereby including more toddlers who were typically developing than could be expected from a random population sample. As the aim of the study was to test the preliminary reliability of the two versions of the screen based on parental understanding of the test items, sample bias may not have affected the results. According to Hyman et al. (2020), an increased rate of 1 in every 59 children is currently diagnosed with autism in the United States of America. No prevalence data are available for South Africa as a result of lack of resources for epidemiological studies (De Vries, 2016).

Similar to the study conducted by Van der Merwe et al. (2017), the test language preference of the participants was leaning towards English. In a multicultural, multilingual country such as South Africa, language proficiency and preference are commonly investigated topics. Posel and Zeller (2016) investigated the change in language use in South Africa from 1996 until 2011 by using the national census results. The study found that English is considered a dominant language in the public office, business and education spheres, including literacy. The Language-in-Education Policy 3(4)(m), National Education Policy, 1996, encourages first language instruction for learners and recommends the acquisition of English as a second language. The research was conducted in a peri-urban area which is part of a large city where English is commonly used (Posel, Hunter, & Rudwick, 2020). The preference for English by participants supports the development of the culturally adapted English version of the M-CHAT-R/F.

The Northern Sotho version of the M-CHAT-R/F was accepted by all participants, even though it was preferred by the minority. Greater support for the use of the Northern Sotho M-CHAT-R/F may be expected in rural areas of South Africa where less prominent use of English is evident (Posel & Zeller, 2016). According to the census, 61% of citizens who identified Northern Sotho as their home language did not have a second language in 2011, with only 19.8% of individuals identifying English as their second language (StatsSA, 2012). Most indigenous African language speakers still prefer to use their home language as it has a ‘symbolic significance as a marker of their identity’ (Posel et al., 2020; Posel & Zeller, 2016). The need for a Northern Sotho translation was confirmed, as 38% of participants indicated that they would rather complete the screening test in Northern Sotho.

### Strengths and limitations

The study results agree with the initial validation study of the M-CHAT-R/F™ (Robins et al., 2014). The sample size, in line with a pilot study, limited the statistical analyses but fulfilled the purpose of determining feasibility of the two versions of the screening test in the current study. Participants indicated a desire for both versions of the South African M-CHAT-R/F.

### Implications or recommendations

The two South African versions of the M-CHAT-R/F are ready for validation which will support early identification of toddlers at-risk for autism in the multicultural and multilingual low- and middle-income country (LMIC) context. Early identification may contribute to earlier diagnosis and intervention. A large-scale validation study is thus recommended before the publication of the instruments.

## Conclusion

The adapted English and Northern Sotho M-CHAT-R/F were shown to be equivalent versions of the M-CHAT-R/F™ in a small-scale pilot study. Preliminary reliability was established. A need for the validation of the Northern Sotho version as well as the adapted English version was identified.

Please contact the first author for access to the two preliminary versions of the M-CHAT-R/F. We would gladly make the two preliminary versions of the M-CHAT-R/F available to readers, but the tests are undergoing further validation and changes may be indicated. As soon as the final versions of the tests become available, the link will be made available to the editor of the SAJCD. It is anticipated that the two versions will eventually be available on the official M-CHAT website.

## Footnotes

1 Northern Sotho is also referred to as Sepedi or Sesotho sa leboa. The correct designation for this language is an ongoing debate of which the authors are aware.
